# Sleep Disturbance, Psychological Distress and Perceived Burden in Female Family Caregivers of Dependent Patients with Dementia: A Case-Control Study

**DOI:** 10.3390/healthcare10122435

**Published:** 2022-12-02

**Authors:** Miguel A. Simón, Ana M. Bueno, Vanessa Blanco, Patricia Otero, Fernando L. Vázquez

**Affiliations:** 1 Health Psychology Research Unit, Department of Psychology, University of A Coruña, 15071 A Coruña, Spain; 2 Department of Evolutionary and Educational Psychology, University of Santiago de Compostela, 15782 Santiago de Compostela, Spain; 3 Department of Clinical Psychology and Psychobiology, University of Santiago de Compostela, 15782 Santiago de Compostela, Spain

**Keywords:** sleep quality, psychological distress, caregiver burden, family caregivers, dementia

## Abstract

This case-control study analyzed the sleep disturbance, psychological distress and perceived burden in female family caregivers of dependent people with dementia (*n* = 74) compared with female family caregivers of dependent people without dementia (*n* = 74) and with age-matched non-caregiver control females (*n* = 74). Participants completed the Pittsburgh Sleep Quality Index (PSQI), the 12-item General Health Questionnaire (GHQ-12), the Caregiver Burden Inventory (CBI) and an ad hoc questionnaire to collect sociodemographic data. There were significant differences between the groups in PSQI total (*F* = 24.93; *p* < 0.001), psychological distress (*F* = 26.71; *p* < 0.001) and in all sleep domains assessed: subjective sleep quality (*F* = 16.19; *p* < 0.001), sleep latency (*F* = 9.5; *p*< 0.001), sleep duration (*F* = 18.57; *p* < 0.001), habitual sleep efficiency (*F* = 19.77; *p* < 0.001), sleep disturbances (*F* = 9.22; *p* < 0.001), use of sleep medications (*F* = 4.24; *p*< 0.01) and daytime dysfunction (*F* = 5.57; *p* < 0.01). In all measures, the female family caregivers of dependent people with dementia showed the significantly higher mean scores. Regarding the two groups of female caregivers, statistically significant differences were found in daily hours of care (*t* = −2.45; *p* < 0.05) and perceived burden (*t* = −3.65; *p* < 0.001), as well as in the following dimensions of caregiver burden: time-dependence burden (*t* = −5.09; *p* < 0.001), developmental burden (*t* = −2.42; *p* < 0.05) and physical burden (*t* = −2.89; *p* < 0.01). These findings suggest that female family caregivers of dependent patients with dementia should be subject to psychopathological screening and preventive cognitive-behavioral interventions in clinical practice in primary health care.

## 1. Introduction

Today, in the European Union more than 9 million people live with different forms of dementia [1] (particularly Alzheimer’s disease), showing a clinical syndrome of chronic progressive cognitive decline and significant functional impairment, one of the leading causes of disability and dependency in the elderly. Although dementia significantly contributes to the institutionalization of older people, most patients with dementia live at home under the care of a family member, mostly a middle-aged or older female [2].

Providing care to dependent persons with dementia is a very demanding task that requires dedication and continuous effort, bringing a set of physical, emotional, financial and social difficulties to the caregivers. This burden associated with caregiving (caregiver burden) negatively affects family caregiver’s health, social and psychological well-being and quality of life, as the extensive research carried out in this field has shown [3,4,5,6]. Many of these negative effects on health are more pronounced in dementia caregivers compared with other groups of family caregivers and with non-caregiver controls [7]. Poor sleep quality and sleep dysfunctions are very usual in dementia caregivers [8] and it is evident that many different factors can contribute. Some of these factors can be directly related to the specific caregiving situation and certain characteristics and clinical aspects of the dementia syndrome, but others may be partially or completely independent. Variables such as age and gender of caregivers are important predisposing factors that may explain changes in different sleep parameters. This fact is relevant, since, as we pointed out earlier, most dementia caregivers are middle-aged or older females.

Previous studies conducted by our research team have shown that caregiver burden is related to poor sleep quality, so that caregivers with high levels of perceived burden have worse sleep quality, compared with both non-caregiver controls (gender and age matched) and family caregivers with low and medium levels of perceived burden [9]. This result is consistent with those reported by Creese et al. [10], Peng et al. [11] and von Känel et al. [12]. Moreover, these family caregivers with high levels of perceived burden showed higher sleep latency and daytime disfunction, more sleep disturbances, higher use of sleep medications and lower sleep duration and sleep efficiency. Likewise, they cared for more people with mental disorders and dedicated more daily hours to the care of dependents.

In addition to the perceived burden, other variables may place an important weight on understanding the negative effects of prolonged and intense care tasks on caregivers’ sleep. In this regard, in more recent subsequent studies we have found a high prevalence of poor sleep quality in family caregivers (76.1%) and poor sleep quality was associated with both higher perceived burden and higher psychological distress [13], obtaining a mean score well above the cutoff score established by the usual screening instruments, such as the 12-item General Health Questionnaire [14]. This finding suggests the existence of high psychopathological morbidity in dependent people’s family caregivers, which has important implications in the field of primary health care.

Following this research line, the purpose of this case-control study was to analyze the sleep disturbance, psychological distress and perceived burden in female family caregivers of dependent patients with dementia compared with female family caregivers of dependent people without dementia and with age-matched non-caregiver control females. It was hypothesized that those females who care for dementia patients will experience more significant negative effects on sleep quality, total sleep time, sleep latency and sleep efficiency, among other important sleep parameters; they will be at an increased psychopathological risk due to higher levels of psychological distress and will show very high levels of perceived burden.

## 2. Materials and Methods

### 2.1. Subjects

The study sample included 148 dependent people’s female family caregivers (74 cared for patients with dementia and 74 cared people without dementia) and 74 age-matched non-caregiver control females. Female family caregivers were randomly recruited from the official register of caregivers of the Dependency Service and Personal Autonomy of the Xunta de Galicia (Autonomous Community of Galicia, Spain). The inclusion criteria to participate in the study were: (a) female gender; (b) being a family caregiver of a dependent person with officially recognized dependence (due to dementia, or due to other physical and mental disorders) and (c) living in the same home with the cared dependent person. The non-caregiver controls, recruited by convenience sampling, were age-matched females who did not provide care to any dependent person. Exclusion criteria of all participants (female family caregivers and female controls) were the presence of any communication problem that could interfere with the usual psychological assessment process and/or having received psychological or psychiatric treatment in the last two months.

The study protocol was conducted in accordance with the Declaration of Helsinki and was reviewed and approved by Bioethics Committee of the University of Santiago de Compostela (Code number 07092016). All subjects (cases and controls) participated completely voluntarily, without receiving any type of incentive, and provided their written informed consent.

### 2.2. Measures

Data on several characteristics of the participants (age, monthly incomes), the care recipients (age) and the care situation (years providing care, daily hours of care) were collected using an ad hoc questionnaire.

The Pittsburgh Sleep Quality Index (PSQI) [15], Spanish version [16], was employed to assess sleep quality and disturbance. This self-report measure comprises 19 items that are combined to form seven component scores (range 0–3): subjective sleep quality, sleep latency, sleep duration, habitual sleep efficiency, sleep disturbances, use of sleep medications and daytime dysfunction. In all cases, higher scores reflected greater difficulties. In addition to the score in each of these subscales, the sum of them generates a single global PSQI score (range 0–21). A global PSQI score above 5 indicates poor sleep quality and pathological difficulties in this area. Using this cutoff score, the PSQI has a diagnostic sensitivity of 89.6% and specificity of 86.5% for distinguishing “good” versus “poor” sleepers and identifying persons with sleep disturbances and difficulties [15]. This Spanish version of the PSQI has favorable psychometric properties, with an appropriate internal consistency, test–retest reliability, convergent validity and predictive value, being a very useful assessment instrument in the field of epidemiological and clinical research [17].

The Spanish version of the 12-item General Health Questionnaire (GHQ-12) [14], carried out by Rocha et al. [18], was utilized to assess psychological distress. This questionnaire is one of the most extensively used screening instruments for psychopathological morbidity and to obtain a general measure of psychological well-being and mental health in epidemiological studies and clinical settings. Using a GHQ-type score (the most suitable when the instrument is used as a one-dimensional screening tool), the total score ranges from 0 to 12, higher scores corresponding to greater psychological distress. The GHQ-12 has displayed acceptable reliability and validity for use in the Spanish population, presenting a high internal consistency both in the general population and in a population over 65 years [18], with a cutoff located at 2/3 to identify the possible presence of psychopathology (sensitivity of 76% and specificity of 80%) [19].

Perceived burden was assessed with the Spanish version of the Caregiver Burden Inventory (CBI) [20], developed by Vázquez et al. [21]. This multidimensional instrument of 24 items has five subscales that explore different dimensions of caregiver burden: time-dependence burden, developmental burden, physical burden, social burden and emotional burden. Briefly, time dependence burden refers to the stress related to the time spent on care tasks and the restriction of the caregiver’s personal time; developmental burden refers to the sense of failure in relation with one’s expectations and intentions; physical burden has to do with physical stress and somatic problems related to care; social burden represents the burden produced in straining to perform roles concerning work or family; finally, emotional burden refers to the negative feelings and embarrassment motivated by the cared people’s behavior. Each item is quantified using a 5-point Likert scale ranging from 0 to 4, so the maximum total score of CBI is 96. Higher scores reflect greater levels of perceived burden; scores above 24 indicate a need for supportive care or some form of respite care, whereas scores above 36 indicate a risk of “burnout”. Therefore, in addition to providing a measure of the impact of the burden on various domains of the caregiver’s life, the CBI allows the obtaining of a general measure of the caregiver’s perceived burden (CBI total score). This Spanish version presents an adequate internal consistency (Cronbach’s *α* = 0.89).

### 2.3. Procedure

With the purpose of standardizing the assessment procedure, a research protocol detailing the objectives, design, participants, measures, instruments, data analysis and ethical issues was developed. In addition, a brief pilot test before starting the study was carried out.

Later, the female family caregivers of dependent people with dementia (Group 1), the female family caregivers of dependent people without dementia (Group 2) and the non-caregiver females group (Control) were contacted through postal mail and phone, explaining the characteristics and interest of the study and encouraging them to participate voluntarily, always ensuring the confidentiality of their responses and the ability to withdraw from the study at any time. Once the informed consent was signed, three experienced clinical psychologists carried out the assessment of the female family caregivers, collecting information on the previously specified characteristics of the participants, the care recipients and the care situation and applying the PSQI, GHQ-12 and CBI. The assessment process was carried out face-to-face in public centers close to the caregivers’ home provided by social community services and was completed in approximately 40 min. Meanwhile, non-caregiver females participated in an online assessment to obtain sociodemographic information and complete the PSQI and the GHQ-12.

### 2.4. Data Analysis

Statistical analyses were conducted using SPSS Statistics 24 (IBM Corp., Armonk, NY, USA). Descriptive statistics, such as the mean and standard deviation for continuous variables and frequency and percentage for categorical variables, were used to summarize the sociodemographic and clinical characteristics of the study groups. The statistical analyses were carried out using one-way analysis of variance (ANOVA) (*F*) and unpaired Student’s *t*-test (*t*) for continuous variables and chi-square test (*χ^2^*) for categorical variables. The homogeneity of variances was tested through the Levene’s test (*L*) and Scheffé’s post-hoc tests for multiple comparisons were conducted. Finally, a multiple linear regression analysis (enter method) was conducted with the purpose of identifying the main associated factors with poor sleep quality in female family caregivers. The significance level was set previously at a *p*-value < 0.05 for all comparisons and all tests were two-tailed.

## 3. Results

The sociodemographic, clinical and care characteristics of the study groups, including the results of statistical analyses, are shown in the Table 1. As summarized in the table, there were no statistically significant differences between the groups in age and monthly incomes. By contrast, significant differences were found in Global PSQI (*F*_2,219_ = 24.93; *p* < 0.001) and psychological distress (GHQ-12) (*F*_2,219_ = 26.71; *p* < 0.001). Concretely, the two groups of female caregivers, very specially the Group 1, presented higher mean scores in these dependent measures than those obtained by the Control Group. The Scheffé’s post-hoc test revealed significant differences between all pairs examined in both variables: Group 1-Group 2, Group 1-Control, Group 2-Control; *p* < 0.05. Before these analyses, the homogeneity of variances was proven by Levene’s test (*L*_2,219_ = 1.17; *p* = 0.31).

**Figure 1:**
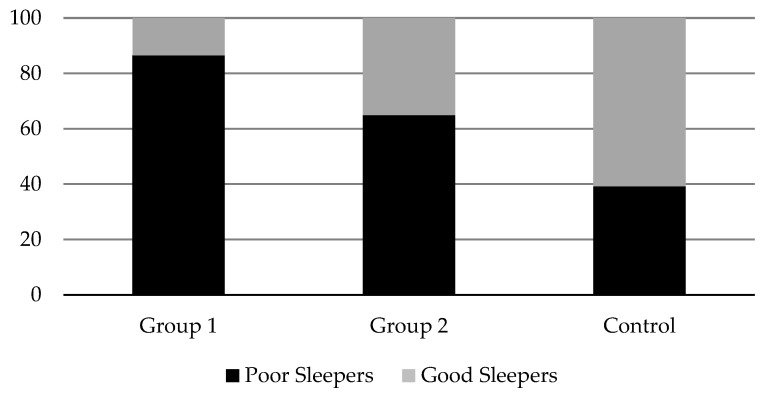
Percentage of “good” and “poor” sleepers in the study groups.

All groups showed poor sleep quality and pathological difficulties in this area (Global PSQI *>* 5), although this was especially pronounced in Group 1. In fact, 86.5% of the subjects in this group reported poor sleep quality, compared to 64.9% and 39.2% observed in Group 2 and Control, respectively (see Figure 1).

Regarding the two groups of female caregivers (Group 1, Group 2), no differences were found either in the age of the care recipient, or in the years providing care. Nevertheless, statistically significant differences were found in daily hours of care (*t* = −2.45; *p* < 0.05) and perceived burden (*t* = −3.65; *p* < 0.001). In both dependent measures, group 1 showed the highest mean scores.

Table 2 presents mean scores and standard deviations obtained by all the study groups in the different PSQI subscales. As seen, significant differences between the groups were found in all subscales, with group 1 showing the highest mean scores. The post-hoc analysis revealed significant differences between all pairs examined in subjective sleep quality and habitual sleep efficiency (Group 1–Group 2, Group 1–Control, Group 2–Control; *p* < 0.05). Moreover, there were significant differences between Group 1–Group 2 and Group 1–Control (*p* < 0.05) in sleep latency, sleep duration, sleep disturbances and daytime disfunction, but not differences were found between Group 2–Control. Finally, with respect to use of sleep medications subscale, only the difference between Group 1–Control was significant (*p* < 0.05).

**Figure 2:**
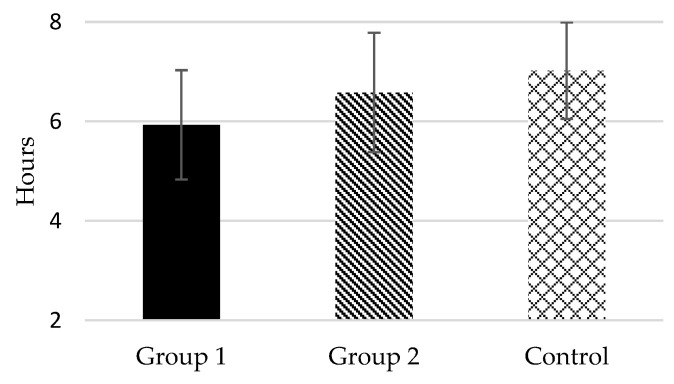
Mean total sleep time of the study groups.

**Figure 3:**
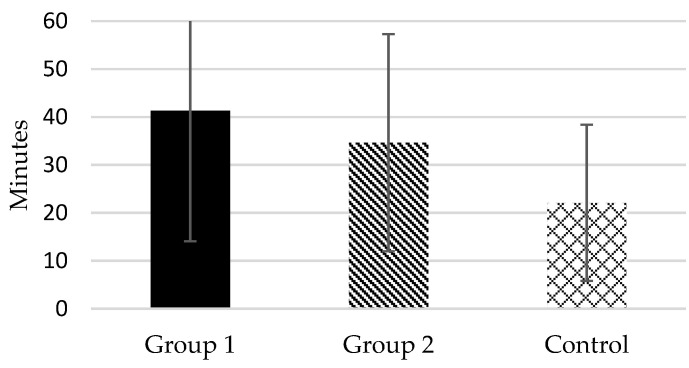
Mean sleep onset latency of the study groups.

According to the responses to item 4 of PSQI, the mean total sleep time of Group 1 was lower than 6 daily hours (5.93 *±* 1.10), whereas in Group 2 and in the Control, it was around 7 h (6.58 *±* 1.20 and 7.02 *±* 0.97, respectively) (see Figure 2). In fact, 74.3% of Group 1 subjects were “short sleepers” (mean total sleep time ≤ 6 h) compared to 45.9% in Group 2 and 21.6% in Control. Likewise, according to the responses to item 2 of PSQI, the mean sleep onset latency of the two groups of female caregivers was higher than 30 min, particularly in Group 1 (41.31 *±* 27.23 in Group 1 and 34.65 *±* 22.65 in Group 2), whereas in the control group it was 22.09 (*±* 16.30) (see Figure 3). As commented, there were significant differences between Group 1–Group 2 and Group 1–Control in these dependent measures, but not differences were found between Group 2–Control.

Mean scores and standard deviations for the five subscales of the CBI are summarized in the Table 3. As appreciated, group 1 showed mean scores higher than group 2 in all subscales. There were statistically significant differences between the groups of female caregivers (Group 1, Group 2) in time-dependence burden (*t* = −5.09; *p* < 0.001), developmental burden (*t* = −2.42; *p* < 0.05) and physical burden (*t* = −2.89; *p* < 0.01), but no differences were found in social burden or in emotional burden.

Finally, a multiple linear regression analysis (enter method) was conducted using sleep quality (global PSQI score) as the dependent variable and age, years providing care, daily hours of care, psychological distress and perceived burden, as independent variables. The analysis revealed that, together, the independent variables explained a significant amount of the variance in the extent of sleep quality (*F*_5,142_ = 22.49; *p* < 0.001; *R^2^* = 0.44; *R^2^_Adjusted_* = 0.42). However, the GHQ-12 score (*β* = 0.52; *p* < 0.001) and the CBI total score (*β* = 0.20; *p* < 0.05) were the only significantly associated variables with sleep quality in female family caregivers.

## 4. Discussion

This case-control study analyzed the sleep disturbance, psychological distress and perceived burden in female family caregivers of dependent patients with dementia compared with female family caregivers of dependent patients without dementia and with age-matched non-caregiver control females. Statistically highly significant differences in Global PSQI and psychological distress were found between the study groups. In both dependent variables, significant differences were found between the female family caregivers of dependent patients with dementia and the non-caregiver females group, between the female family caregivers of dependent patients without dementia and the non-caregiver females group, but also between the two groups of female family caregivers. Compared with female family caregivers caring for patients without dementia, those who take care of patients with dementia displayed poorer sleep quality (prevalence of poor sleep quality above 86%) and the highest levels of psychological distress, well above the cutoff score established to identify psychopathology. Moreover, according to the initially raised hypotheses, dementia caregivers showed increased onset sleep latency (greater than 40 min), shorter sleep duration, more reduced habitual sleep efficiency and more daytime disfunction and sleep disturbances. Mean total sleep time in this group was lower than 6 daily hours (74.3% of its members being “short sleepers”), which is clearly inappropriate for health maintenance in adults [22]. Use of sleep medications was the only variable in which no significant differences were found between the two groups of female family caregivers, although they were found between dementia caregivers and controls. By contrast, no differences were found between female family caregivers of dependent patients without dementia and the non-caregiver females group in the following sleep parameters: sleep latency, sleep duration, sleep disturbances and daytime disfunction.

These findings are consistent with previous studies that have noted more negative effects on sleep and psychological well-being in dementia caregivers that in other groups of family caregivers [7,23,24], probably because certain clinical aspects of the dementia syndrome (such as nighttime awakenings, misidentification of close relatives, agitation and so on) may increase the vulnerability of the female caregivers and make them particularly prone to psychological distress [25]. In fact, the high prevalence of poor sleep quality found in our study (86.5%) is very similar to that detected by other authors. This is the case, for example, of the study carried out by Peng et al. [11] with the purpose of identifying factors associated with sleep in family caregivers of individuals with dementia, whose findings indicated a prevalence of poor sleep quality of 91.7%. Moreover, in our study, the finding of short sleep time showed by dementia caregivers is very important and reveals that these female caregivers may be at increased risk for negative health consequences such as decline in cognitive and functional abilities, cardiovascular diseases, metabolic dysfunctions and so on [26,27].

Regarding the two groups of female family caregivers, significant differences were found in daily hours of care (but not in the age of the care recipient or in the years providing care) and in the total score of perceived burden. In both measures, female family caregivers of dependent patients with dementia showed significantly higher values. The total score of perceived burden in this group was very high, far exceeding the established threshold that indicates risk of “burnout”. Regarding the several dimensions of the perceived burden, the differences between the groups of female caregivers were limited to time-dependence burden, developmental burden and physical burden. Differences related both to the time spent on care tasks and the restriction of the caregiver’s personal time, as well as to the sense of failure in relation with the one’s expectations and to the physical stress and somatic problems related to care. The high levels of burden identified suggest that formal support networks for caregivers of dementia patients should be established and the provided services should be designed to target different types of burdens and the factors contributing to their development and maintenance [19,28,29].

In this study, several limitations need to be noted. Specifically, this is a cross-sectional study, not longitudinal, so that it does not allow an investigation of the evolution and changes at different time points. Besides, due to the large size of the sample studied, sleep parameters have been measured through self-report questionnaire without using objective measures (such as polysomnography), collecting differently data from female caregivers and controls (i.e., online vs. face to face), and this could have exerted a minor impact on the results. Likewise, no more specific information was obtained regarding circadian rhythms and sleep hygiene, which are important factors that can contribute to the development and maintenance of sleep dysfunctions [30]. Despite these limitations, this study included reliable, valid and widely used assessment procedures in clinical research and the findings obtained reveal the need to implement screening programs for the early detection of psychological dysfunctions and sleep disturbances in primary health care of female caregivers of dependent patients with dementia, as well as to develop specific cognitive-behavioral treatment programs focused to this collective for the effective and efficient management of these health problems when they occur. This is the priority objective of our future research in this field.

## 5. Conclusions

The results of this study allow us to conclude that female family caregivers of dependent patients with dementia show more sleep disturbance, psychological distress and perceived burden compared to female family caregivers of dependent patients without dementia and age-matched female controls. Specifically, our findings indicated a high prevalence of poor sleep quality in dementia caregivers, as well as marked impairment in all sleep domains evaluated. Furthermore, our findings also discovered that psychological well-being of these female caregivers is severely affected, presenting a high risk of psychopathological morbidity. For this reason, our future research will be focused on the development and evaluation of treatment programs for the management of these health problems in dementia caregivers.

## Figures and Tables

**Table 1:** Sociodemographic, clinical and care characteristics of the study groups.

	Group 1 (*n* = 74)	Group 2 (*n* = 74)	Control (*n* = 74)	Comparison
Age (years)	56.52 (9.65)	58.02 (7.09)	56.42 (10.43)	*F*_2,219_ = 0.656; *p* = 0.520
Monthly incomes (Euros) <1000 1000–2000 >2000 Do not know/ No answer	16 (21.6%) 29 (39.2%) 8 (10.8%) 21 (28.4%)	13 (17.6%) 34 (45.9%) 4 (5.4%) 23 (31.1%)	15 (20.3%) 30 (40.5%) 7 (9.5%) 22 (29.7%)	*χ^2^* = 2.23; 6 *df*; *p* = 0.897
Global PSQI	10.42 (4.13)	7.58 (3.99)	5.88 (3.71)	*F*_2,219_ = 24.93; *p* < 0.001 *
Psychological distress (GHQ-12)	4.98 (3.20)	3.64 (3.05)	1.45 (2.60)	*F*_2,219_ = 26.71; *p* < 0.001 *
Age (years) of the care recipient	71.31 (21.87)	75.15 (20.48)		*t* = 1.101; 146 *df*; *p* = 0.273
Years providing care	13.96 (10.48)	13.94 (10.79)		*t* = −0.010; 146 *df*; *p* = 0.992
Daily hours of care	17.35 (4.90)	15.37 (4.92)		*t* = −2.45; 146 *df*; *p* < 0.05 *
Perceived burden (CBI total score)	45.47 (13.62)	36.88 (14.96)		*t* = −3.65; 146 *df*; *p* < 0.001 *

Note: data are expressed as frequency (*n*) and percentage (%) for categorical variables and as mean ($\bar{X}$) and standard deviation (*SD*) for continuous variables. Group 1: female family caregivers of dependent people with dementia; Group 2: female family caregivers of dependent people without dementia; Control: non-caregiver females group. PSQI: Pittsburgh Sleep Quality Index; GHQ-12: 12-item General Health Questionnaire; CBI: Caregiver Burden Inventory. * Significant difference.

**Table 2:** Mean scores (standard deviations) for subscales of PSQI in all the study groups.

PSQI Subscales	Group 1 (*n* = 74)	Group 2 (*n* = 74)	Control (*n* = 74)	Comparison
Subjective sleep quality	1.61 (0.80)	1.24 (0.71)	0.91 (0.72)	*F*_2,219_ = 16.19; *p*< 0.001
Sleep latency	1.87 (1.08)	1.36 (1.20)	1.12 (0.90)	*F*_2,219_ = 9.5; *p*< 0.001
Sleep duration	1.58 (0.81)	1.10 (0.89)	0.80 (0.64)	*F*_2,219_ = 18.57; *p*< 0.001
Habitual sleep efficiency	1.46 (1.17)	1.03 (1.08)	0.41 (0.77)	*F*_2,219_ = 19.77; *p*< 0.001
Sleep disturbances	1.60 (0.59)	1.22 (0.58)	1.26 (0.62)	*F*_2,219_ = 9.22; *p*< 0.001
Use of sleep medications	0.89 (1.25)	0.53 (1.08)	0.38 (0.94)	*F*_2,219_ = 4.24; *p*< 0.01
Daytime dysfunction	1.45 (0.86)	1.11 (0.83)	1.01 (0.78)	*F*_2,219_ = 5.57; *p*< 0.01

Note: all values are significant.

**Table 3:** Mean scores (standard deviations) for subscales of CBI.

CBI Subscales	Group 1 (*n* = 74)	Group 2 (*n* = 74)	Comparison
Time-dependence burden	17.70 (2.86)	15.26 (2.98)	*t* = −5.09; 146 *df*; *p* < 0.001 *
Developmental burden	10.32 (5.39)	8.30 (4.80)	*t* = −2.42; 146 *df*; *p* < 0.05 *
Physical burden	8.84 (3.93)	6.88 (4.29)	*t* = −2.89; 146 *df*; *p* < 0.01 *
Social burden	5.89 (3.39)	4.54 (3.56)	*t* = −1.83; 146 *df*; *p* = 0.069
Emotional burden	2.69 (2.47)	1.93 (1.71)	*t* = −1.47; 146 *df*; *p* = 0.143

* Significant difference.

## Data Availability

The data presented in this study are available on request from the corresponding author. The data are not publicly available due to confidentiality issues.
